# Focused Patterning of Electrospun Nanofibers Using a Dielectric Guide Structure

**DOI:** 10.3390/polym13091505

**Published:** 2021-05-07

**Authors:** Byeongjun Lee, Younghyeon Song, Chan Park, Jungmin Kim, Jeongbeom Kang, Haran Lee, Jongwon Yoon, Seongjin Cho

**Affiliations:** School of Mechanical Engineering, Chungnam National University, 99 Daehak-Ro, Yuseong-Gu, Daejeon 34134, Korea; michael489@naver.com (B.L.); yh9333@daum.net (Y.S.); cksdl4608@naver.com (C.P.); kjm5301@naver.com (J.K.); wjdqjarkd@naver.com (J.K.); ktten55@naver.com (H.L.); jongwon3498@naver.com (J.Y.)

**Keywords:** electrospinning, dielectric, electric field, nanofiber, patterning

## Abstract

The patterning of electrospun fibers is a key technology applicable to various fields. This study reports a novel focused patterning method for electrospun nanofibers that uses a cylindrical dielectric guide. The finite elements method (FEM) was used to analyze the electric field focusing phenomenon and ground its explanation in established theory. The horizontal and vertical electric field strengths in the simulation are shown to be key factors in determining the spatial distribution of nanofibers. The experimental results demonstrate a relationship between the size of the cylindrical dielectric guide and that of the electrospun area accumulated in the collector. By concentrating the electric field, we were able to fabricate a pattern of less than 6 mm. The demonstration of continuous line and square patterning shows that the electrospun area can be well controlled. This novel patterning method can be used in a variety of applications, such as sensors, biomedical devices, batteries, and composites.

## 1. Introduction

Electrospinning is one of the simplest and most effective ways to fabricate nanofibers. These nanofibers have potential applications in many fields, such as nanocatalysis, filtration, tissue scaffolding, textile fabric, fibrous porous media, drug delivery, and composites [1,2,3,4,5,6,7,8,9,10]. In particular, membranes manufactured using electrospinning have the advantages of large specific surface area, high mechanical stiffness, and good insulation. Therefore, such membranes are widely used in flexible insulating films [11,12] sensors [13,14], gas sensing devices [15], and batteries [16,17,18].

Because of the chaotic whipping nature of electrospinning, electrospun fibers are typically deposited as a randomly oriented mat on a conductive collector. Many efforts have been undertaken to control the resulting fiber pattern by either controlling the electric field or changing the architecture of the collector. Various methods can be used to control the electric field under which nanofibers are electrospun; parallel electrodes [19,20,21,22], an auxiliary electrode [23,24,25], a ring collector [26], or a side-wall electrode [27] can be fabricated from shaped continuous nanofibers. These studies use the interaction between the spinning jet and the electrostatic repulsion to control the behavior of the fiber and pattern the fiber. However, this approach increases the size of the electrospinning setup, due to additional electrode configuration. Conversely, fiber pattern changes can also be achieved by changing the collector architecture. Previous studies using prepatterned collectors have been obtain geometric shape of patterned fiber mat. However, the shape of the fibers depends on prepatterned collectors and masks. This means that a single pattern collector can produce only a single shape, so a new collector can be constructed for each new shape to be crafted from nanofibers. The fibers are then guided to the new collector, whether it is an insulating film [11,12], a rotating drum [28,29,30,31], a disk collector [32], a rotating water collector [33], or a metal mesh [34].

Here, we report a new patterning method for nanofibers using a cylindrical dielectric guide. We controlled and focused the electric field of the electrospinning process using a dielectric structure without any additional electrode configuration. The new patterning method developed can miniaturize the electrospinning setup, which is a limitation of previous studies, and is not dependent on prepatterned collectors and masks. In this study we analyzed the electric field focusing phenomenon using the finite elements method (FEM), which is a numerical technique used to discover solutions of incomplete differential condition equations and ground its explanation in established theory. Finally, we successfully patterned nanofibers using a cylindrical dielectric guide into the desired shape using a motorized stage. We believe that the advantages of the method described herein can be extended to tissue engineering, drug delivery, sensor technology, and surface modification. We expect that this approach can be used not only for filtration and fabrication of reinforcing composites but also with other functional materials.

## 2. Materials and Methods

### 2.1. Materials

We fabricated nanofibers using various polymers (polyacrylonitrile, PAN; polyethylene oxide, PEO; and polyurethane, PU) to confirm the patterning effect of cylindrical dielectric guides. PU, PAN, and PEO are widely known as polymers that are generally used for electrospinning, and they use different solvents such as water and organic solvent. A solution of PU (Pellethane 2363-80AE, Lubrizol, Wickliffe, OH, USA) in tetrahydrofuran (THF) and dimethylformamide (DMF) was synthesized at a DMF:THF ratio of 60:40 at room temperature (20 °C–25 °C) for 24 h. A solution of PEO (average M_v_ ≈ 300,000) in distilled water (DI water) was synthesized at room temperature for 24 h. A solution of PAN (average M_v_ ≈ 1,300,000) in ethanol was synthesized at room temperature for 24 h. These three solutions were blended at a ratio of 14 wt%, 8 wt%, and 12 wt%, respectively. All materials other than the PU were purchased from Sigma-Aldrich (St. Louis, MO, USA).

### 2.2. Electrospinning Setup with Cylindrical Dielectric Guides

The cylindrical dielectric guide in the electrospinning system was made of polylactic acid (PLA) using a fuse deposition modeling method 3D printer (CUBICON Single Plus, Cubicon, Seongnam, Korea). By using a lamination method 3D printing is made economical and it has a high degree of freedom in manufacturing shapes. In this study, cylinder structures of various sizes were fabricated with a 3D printer to concentrate the electric field. The cylinder structure is symmetrical in the axial direction and can generate a uniform electric field.

In addition, PLA was selected as the material for producing the cylindrical dielectric guide. PLA is widely used as a material for 3D printing due to its good formability and low shrinkage of the product. Cylindrical dielectric guides were manufactured with diameters (*D_guide_*) ranging from 30 to 120 mm. Each mixed solution was loaded into a 3-mL syringe with a 21-gage needle for electrospinning. The syringe was placed in a syringe pump (Legato 100 syringe pump, KD Scientific, Holliston, MA, USA), and the solution was pulled out at a rate of 1 mL/h from the needle tip. The tip of the syringe was connected to the positive electrode of a high-voltage power supply (ERP series, Matsusada Precision, Kusatsu, Japan). Each mixed solution was electrospun onto an aluminum plate located 75 mm from the needle tip. The cylindrical dielectric guide was located at the center of the syringe tip.

### 2.3. Nanofiber Analysis

**Figure 1:**
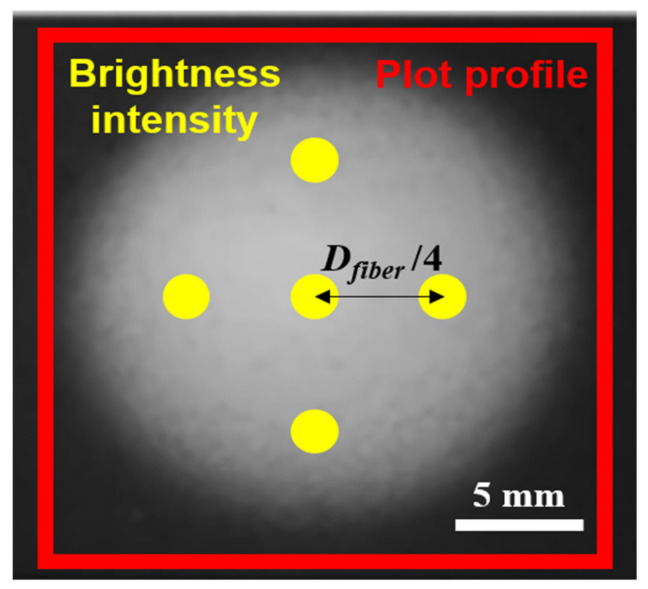
Brightness intensity analysis points and plot profile.

The morphology and diameter of each nanofiber were determined with an FE-scanning electron microscope (SEM; Model S-4800/SU8230, Hitachi, Tokyo, Japan). An SPT-20 unit (COSEM, Daejeon, Korea) operating at 500 mA for 120 s was used to sputter-coat nanofibers with platinum. An optical camera (EOS 800D, Canon, Tokyo, Japan) and ImageJ software (National Institutes of Health, Bethesda, MD, USA) were used to analyze the electrospun nanofibers. Figure 1 shows the plot profile and brightness intensity analysis points used by ImageJ software to analyze the density and uniformity of fabricated nanofibers. Brightness intensity analysis was conducted at five points: at the center of the electrospun nanofiber and at four cardinal points located at a radius of one-quarter of the nanofiber diameter.

### 2.4. Electric Field Simulation

**Figure 2:**
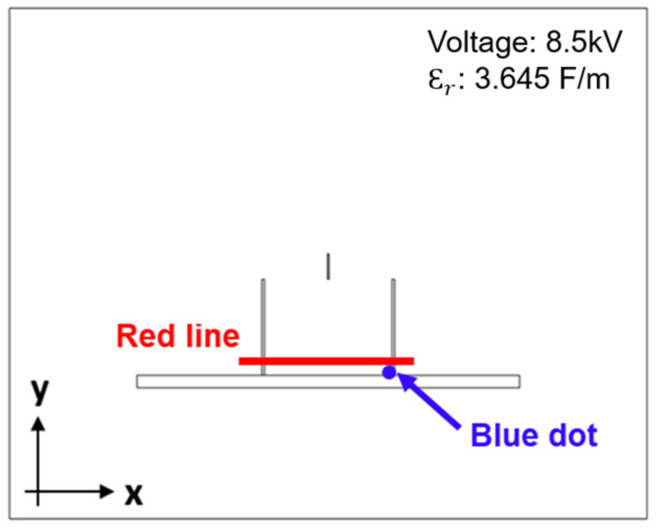
Electric field analysis based on the red line and blue dot.

Electrostatic forces are the dominant forces applied to the jet, so the distribution and strength of the electric field greatly affect the trajectory of the jet. Analyzing the electric field provides a better understanding of the effects of jet path control modifications. The distribution and strength of the electric field in the plane of the electrospinning setup were analyzed using the finite elements method (FEM). FEM is a numerical technique which is utilized to discover solutions of incomplete differential condition equations (PDE). It gives an option approach to see high-electric field. Since, the practical dimensions and material properties can be used for the FEM calculation, it allows us to envision the electric field profile and to comprehend the impacts of material characteristics, dimension and physical geometry [35]. The distribution and strength of the electric field in the plane of the electrospinning setup were calculated using the electrostatics module (2D) in COMSOL Multiphysics (COMSOL Inc., Stockholm, Sweden) software. To build a model in software, PLA (ε_r_ = 3.645) was used for the cylindrical dielectric guide. Aluminum was used as the collector in the building of the model, and the analysis was conducted with a very fine mesh size (minimum element size: 3.75 × 10^−5^ m) under air. As for the electrical conditions, a voltage of 8.5 kV was applied to the needle, and the collector was grounded. The red line in Figure 2 shows the region used by COMSOL Multiphysics to analyze the electric field strength in the X- and Y-directions near the collector. The blue dot shows the region used by COMSOL Multiphysics to analyze the electric field strength in the X-direction at the bottom part of the cylindrical dielectric guide.

### 2.5. Patterning of Nanofibers

For continuous patterning of nanofibers, a setup differing from a conventional electrospinning setup was required. A 1-axis stage (V-528, PI, Karlsruhe, Germany) and rotating structure was placed at the bottom of the collector. For patterning, the collector was fixed on the 1-axis stage. A cylindrical dielectric guide with a *D_guide_* between 30 and 120 mm was placed between the needle and the collector. Its placement 1 mm from the collector ensured that the cylindrical dielectric guide would experience no interference due to movement of the collector. The positive voltage generated by the high-voltage power supply was applied to the needle, and the negative voltage was applied to the collector. The setup was grounded in order to prevent charge from accumulating in the nanofibers. The stage was driven by computer control software (PiMikroMove and LabVIEW), and the solution was electrospun on the moving collector. Computer control software was coded in hold, line, and square patterns and operated in a variety of shapes.

## 3. Results and Discussion

### 3.1. Electrospinning Setup

**Figure 3:**
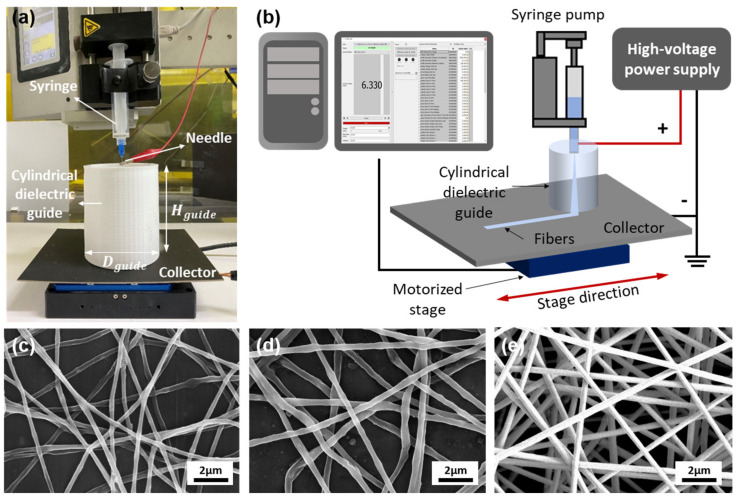
(**a**) Focused patterning setup with cylindrical dielectric guide; (**b**) electrospinning schematic; and (**c**–**e**) SEM images of (**c**) polyacrylonitrile (PAN); (**d**) polyethylene oxide (PEO); and (**e**) polyurethane (PU) nanofibers.

Figure 3a shows a hollow cylindrical dielectric guide fabricated from PLA (ε_r_ = 3.645 F/m) with an average diameter (*D_guide_*) of 30–120 mm and a height of 75 mm (*H_guide_*) located between a needle and the collector. The needle tip is placed in the center of the cylindrical dielectric guide. For the PU, PEO, and PAN solutions, voltages of 8.5, 10.0, and 9.7 kV were respectively applied to the needle tip through the high-voltage power supply. The negative electrode was connected to the collector, where nanofibers were deposited. The grounding of the collector prevented the accumulation of electric charge on the nanofibers electrospun on the collector. The stage was controlled at a rate of 1 mm/s, moving in a straight line; the movement range was 50 mm (Figure 3b). Figure 3c–e shows SEM images of electrospun PAN, PEO, and PU nanofibers. Each nanofiber had a diameter of 300–500 nm, forming a matrix between fibers without bead-shaped defects. All three polymers were successfully electrospun using a cylindrical dielectric guide.

### 3.2. Analysis of Electric Field with Cylindrical Dielectric Guides

**Figure 4:**
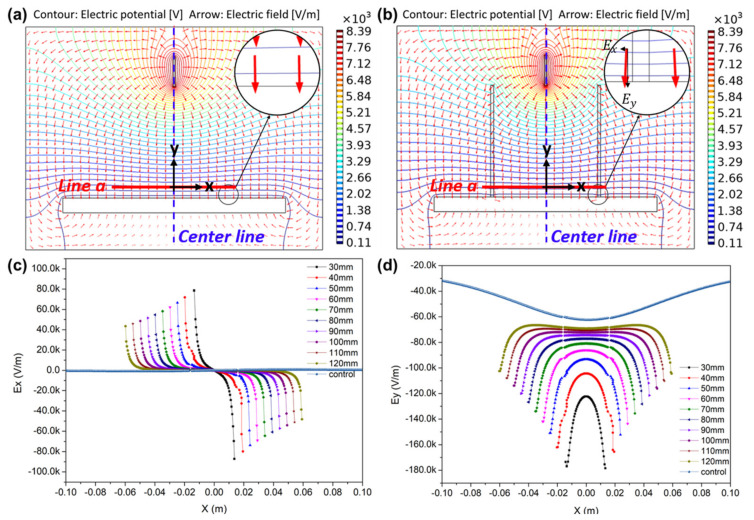
(**a**,**b**) Simulated electric potential (contour in figure) and electric field (arrow in figure) distribution during electrospinning (**a**) without and (**b**) with a cylindrical dielectric guide and (**c**,**d**) electric field strength (**c**) *Ex* and (**d**) *Ey* along reference *Line a* for various cylindrical dielectric guide diameters (Control in Figure 4c,d mean without cylindrical dielectric guide).

Figure 4a,b shows the predicted electric field distribution for two cases of a conventional electrospinning setup: without and with a cylindrical dielectric guide. As shown in Figure 4a, the electrical field of a conventional electrospinning setup without a cylindrical dielectric guide showed a uniform distribution at *Line a*, lying at a distance of 1 mm from the collector. However, the electric field line in Figure 4b shows greater convergence to the center line than that in Figure 4a. Thus, the electric field diverges at the top of the cylindrical dielectric guide but converges to the center line at the bottom. This observation can be confirmed using the *E_x_* and *E_y_*.

Figure 4c,d shows the electric field *E_x_* and *E_y_* at *Line a*. Figure 4c depicts *E_x_* at *Line a*, which amounts to a uniform control line when there is no cylindrical dielectric guide. However, when there is a cylindrical dielectric guide, *E_x_* increases in the direction from the cylindrical dielectric guide to the center line. This means that electrostatic force is applied horizontally to the nanofibers during electrospinning, and fibers are focused in the direction of the center line. The smaller the *D_guide_*, the larger the *E_x_* is applied to nanofibers, increasing from 40 to 100 kV/m. As a result, the smaller the *D_guide_*, the greater the force that can be applied to focus fibers toward the center line. Figure 4d shows *E_y_* applied to nanofibers at *Line a*. The smaller the *D_guide_*, the stronger the attraction of the nanofibers to the collector direction at a maximum of 180 kV/m, causing a decrease in the diameter of deposited nanofibers. Conversely, as the *D_guide_* increases, *E_y_* becomes distributed uniformly and widely within cylindrical dielectric guides. As a result, the distribution of nanofibers is determined using *E_x_* and *E_y_*. The smaller the *D_guide_*, the smaller the area into which the nanofibers are collected. The larger the *D_guide_*, the larger the area into which the nanofibers are collected.

### 3.3. Analysis of Focused Patterned Nanofibers

**Figure 5:**
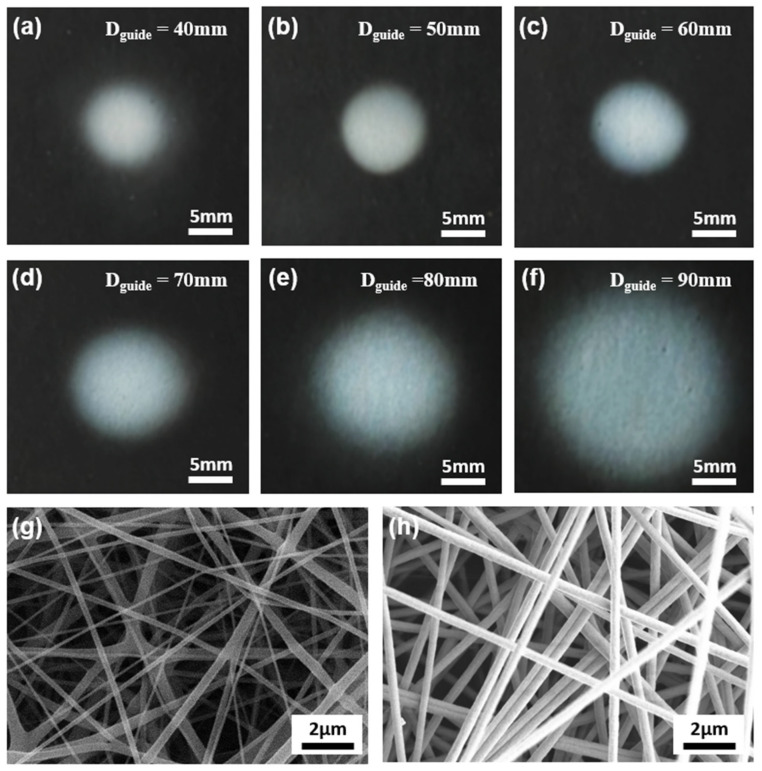
(**a**–**f**) Optical images of electrospun nanofiber mats of diameters (**a**) 90, (**b**) 80, (**c**) 70, (**d**) 60, (**e**) 50, and (**f**) 40 mm; (**g**,**h**) SEM images of PU nanofibers electrospun using cylindrical dielectric guides of diameters (**g**) 90 and (**h**) 40 mm.

To confirm the patterning effect of the dielectric guide, we fabricated PU nanofibers using various guide diameters. When a high voltage was applied to the needle tip, the jet erupted from the tip of the spinneret and moved to the collection plate. A cylindrical dielectric guide was placed between the needle tip and the collector. Figure 5a–f shows optical images of PU nanofibers electrospun using cylindrical dielectric guides with *D_guide_* ranging from 40 to 90 mm. These images show that as *D_guide_* decreased from 90 to 40 mm, the average diameter of the electrospun area (*D_fiber_*) decreased by approximately 80%, from 50 to 13 mm. In addition, the density of the electrospun nanofibers increased as *D_guide_* decreased. This result shares an explanation with the electric field simulation result: *E_x_* repels nanofibers increasingly strongly from the inner wall of the cylindrical dielectric guide as *D_guide_* decreases. We provide a more detailed quantitative analysis of this result in the next paragraph. Figure 5g,h shows SEM images of PU nanofibers with *D_guide_* of 90 and 40 mm, respectively. The “analyze particles” tool of Image J was used to calculate the nanofiber density. In the case of a nanofiber with a *D_guide_* of 90 mm, pores comprised 27.9% of the total area, whereas in the case of a nanofiber with a *D_guide_* of 40 mm, pores comprised only 11.0%. Thus, the smaller the *D_guide_*, the higher the fiber density.

**Figure 6:**
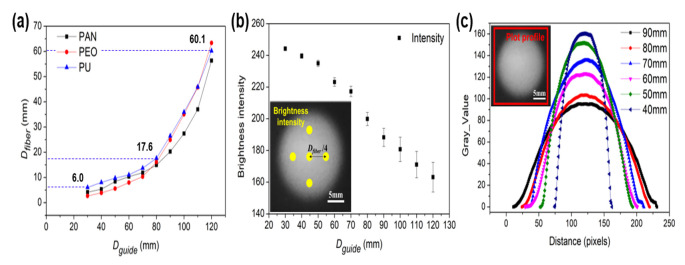
(**a**) Diameters of electrospun PAN, PEO, and PU nanofiber mats as functions of cylindrical dielectric guide diameter; (**b**) brightness intensity as a function of cylindrical dielectric guide diameter; (**c**) fiber shape expressed in gray value.

Figure 6a shows trends in average *D_guide_* for nanofibers electrospun from each of the three solutions as functions of *D_guide_*. Focused patterning fibers exhibited *D_fiber_* of 6–60 mm. At a *D_guide_* of 120 mm, a nanofiber with a *D_fiber_* of 60.1 mm was fabricated, i.e., approximately half of *D_guide_*. At a *D_guide_* of 30 mm, a nanofiber with a *D_fiber_* of 6 mm was fabricated, i.e., approximately 1/5 of *D_guide_*. *D_fiber_* decreased exponentially as *D_guide_* decreased, because the smaller the *D_guide_*, the stronger the *E_x_* was, as shown in Figure 4c. As a result, the smaller the *D_guide_*, the more focused the electric field became, causing a nonlinear decrease in *D_fiber_*. However, as *D_guide_* decreased, *E_y_* decreased, leading to the adverse effect of bead formation.

Figure 6b shows brightness intensity as a function of *D_guide_*. The collector used in the experiment had an RGB value close to 0; as nanofibers accumulated, the RGB value approached the maximum value of 255. The relative density of nanofibers was estimated accordingly. The brightness intensity of nanofibers was measured at five points: at the center of the fiber *D_fiber_* and at four cardinal points located at a radius of one-quarter of the nanofiber diameter. Brightness intensity reached its minimum of 163.1 at a *D_guide_* of 120 mm; brightness intensity was 239.6 at a *D_guide_* of 30 mm, i.e., ~1.5 times the minimum value. This means that as *D_guide_* decreased, the nanofiber density increased. The standard deviation of brightness intensity among the five points measured was 1.5 at a *D_guide_* of 120 mm but 9.4 at a *D_guide_* of 30 mm, i.e., a factor of 6.3 difference. This means that the smaller the *D_guide_*, the greater the even density distribution of nanofiber.

Figure 6c is a plot profile graph showing the distribution of fibers measured in pixel units. In the graph, the maximum gray values of the fibers range over approximately a factor of 2, from 89 to 178, which can be interpreted as representing a factor of 2 difference in thickness at the center of the nanofiber collection area. At small *D_guide_*, parabola-shaped mats of nanofibers were formed, with large differences in thickness between the center and edge of the nanofiber collection area. As *D_guide_* increased, nanofiber mats of more uniform thickness were formed. Thus, it was possible to selectively apply fibers to achieve either uniform application surfaces or thickly matted centers. 

### 3.4. Electrospun Patterned Nanofibers

**Figure 7:**
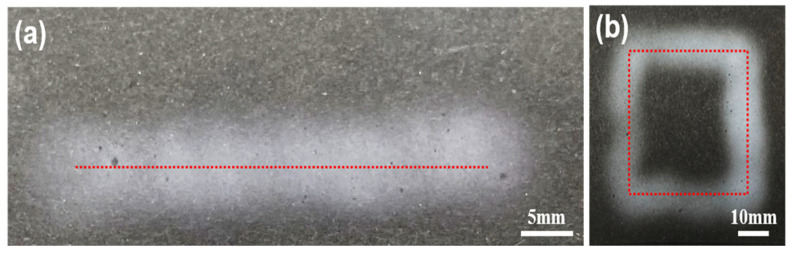
Electrospun nanofibers in (**a**) a line pattern and (**b**) a square pattern.

Figure 7 shows line and square patterns of PU nanofibers fabricated using a cylindrical dielectric guide. Both patterned nanofibers were fabricated using a cylindrical dielectric guide with a *D_guide_* of 60 mm and a 1-axis stage. Nanofibers were electrospun into the cylindrical dielectric guide from a needle tip to which a voltage of 8 kV was applied, and the collector fixed on the stage moved at a rate of 1 mm/s. Figure 7a shows the fabricated nanofibers; the stage moved along the red line to form the fiber pattern. Figure 7b shows the nanofibers patterned in a square; the line width here is similar to that in the line pattern. Each electrospun nanofiber maintained a *D_fiber_* of 10 mm and was continuously electrospun along a stage length of 50 mm. This method has the advantageous capability to pattern nanofibers in a desired shape through computer control software.

## 4. Conclusions

In this study, we verified that it is possible to pattern nanofibers by using a cylindrical dielectric guide. The cylindrical dielectric guide focused the electric field in the space in which electrospinning occurs, and the electrospun nanofibers were able to be patterned without an additional power source. Electric field analysis in COMSOL Multiphysics confirmed that nanofiber electrospinning inside the cylindrical dielectric guide experienced an electrical force applied in the direction from the inner wall of the cylindrical dielectric guide toward the central line. Experimental results compared well with simulation results. We also demonstrated the use of a cylindrical dielectric guide to produce continuous line and square patterns and to control nanofiber mat diameter in the range of 6–60 mm. Cylindrical dielectric guides for patterning electrospun nanofibers are expected to be utilized as a new, simpler diameter control method in sensor packaging, gas sensing, tissue scaffolding, and the composites industry.
